# Bayesian analysis of a mastitis control plan to investigate the influence of veterinary prior beliefs on clinical interpretation

**DOI:** 10.1016/j.prevetmed.2009.05.029

**Published:** 2009-10-01

**Authors:** M.J. Green, W.J. Browne, L.E. Green, A.J. Bradley, K.A. Leach, J.E. Breen, G.F. Medley

**Affiliations:** a School of Veterinary Medicine and Science, University of Nottingham, Sutton Bonington Campus, Sutton Bonington LE12 5RD, UK; b School of Mathematical Sciences, University of Nottingham, Nottingham NG7 2RD, UK; c Department of Biological Sciences, University of Warwick, Coventry CV4 7AL, UK; d Department of Clinical Veterinary Science, University of Bristol, Langford House, Langford, Bristol BS40 5DT, UK

**Keywords:** Prior distribution, Clinical decision making, Bayesian analysis, Mastitis

## Abstract

The fundamental objective for health research is to determine whether changes should be made to clinical decisions. Decisions made by veterinary surgeons in the light of new research evidence are known to be influenced by their prior beliefs, especially their initial opinions about the plausibility of possible results. In this paper, clinical trial results for a bovine mastitis control plan were evaluated within a Bayesian context, to incorporate a community of prior distributions that represented a spectrum of clinical prior beliefs. The aim was to quantify the effect of veterinary surgeons’ initial viewpoints on the interpretation of the trial results.

A Bayesian analysis was conducted using Markov chain Monte Carlo procedures. Stochastic models included a financial cost attributed to a change in clinical mastitis following implementation of the control plan. Prior distributions were incorporated that covered a realistic range of possible clinical viewpoints, including scepticism, enthusiasm and uncertainty. Posterior distributions revealed important differences in the financial gain that clinicians with different starting viewpoints would anticipate from the mastitis control plan, given the actual research results. For example, a severe sceptic would ascribe a probability of 0.50 for a return of <£5 per cow in an average herd that implemented the plan, whereas an enthusiast would ascribe this probability for a return of >£20 per cow. Simulations using increased trial sizes indicated that if the original study was four times as large, an initial sceptic would be more convinced about the efficacy of the control plan but would still anticipate less financial return than an initial enthusiast would anticipate after the original study. In conclusion, it is possible to estimate how clinicians’ prior beliefs influence their interpretation of research evidence. Further research on the extent to which different interpretations of evidence result in changes to clinical practice would be worthwhile.

## 1 Introduction

Much research in health aims to understand the processes related to disease, and, ultimately, to improve health interventions in populations and clinical management of individual patients. Results from such research are of greatest value when findings translate into improvements in health and welfare. For new research to be worthwhile in terms of changing clinical decisions, the clinical beliefs (‘clinical priors’) of the decision-makers need to be understood and taken into account. This is important because the inferences made by each individual in the light of a particular clinical trial will be influenced by their prior beliefs or degree of scepticism about the plausibility of different possible results (Chaloner and Rhame, 2001; Spiegelhalter et al., 2004a). Whilst it is important to note that an individual clinician's decision to change their approach to disease control is complex (and may depend on a variety of psychological and circumstantial factors, as well as the perceived cost or health benefit (Crosskerry, 2005)), investigation of the influence of prior beliefs is an important element of understanding this decision process.

The fact that there are prior beliefs in medicine is well documented (e.g. Fallowfield et al., 1997; Peto and Baigent, 1998; Spiegelhalter et al., 2004a; Henry et al., 2006). Such beliefs have been used to predict and understand the decision making process of medical physicians (Parmar et al., 1994; Brophy and Joseph, 1995; Harrell and Shih, 2001). Documenting prior beliefs before undertaking clinical trials is useful to gauge the likely response of clinicians to possible trial outcomes (and therefore whether clinical decision-making will change) and also to aid in sample size calculations—the strength of evidence required differs depending on the degree of scepticism at the outset (Kadane, 1994; Parmar et al., 1994; Chaloner and Rhame, 2001). However, prior distributions are not necessarily pre-specified or unique and can be used *post hoc*, to assess how research results may be interpreted, conditional on varying personal viewpoints (Spiegelhalter et al., 2004a).

In veterinary medicine, little is known of the heterogeneity in clinical beliefs of veterinary surgeons, although this will fundamentally affect interpretation of research evidence and approaches to disease management. The use of a ‘community of priors’ has been proposed by Kass and Greenhouse (1989) to describe a spectrum of realistic viewpoints that should be considered when interpreting new evidence.

Bayesian methods are particularly suited to the incorporation of prior beliefs in a probabilistic decision-theoretic context (Berry and Stangl, 1996; O’Hagan and Luce, 2003) and have some advantages over frequentist approaches that include the following, a straightforward framework for predicting future events and the ability to include information, with associated uncertainty, from a variety of sources (Berry, 1993; Spiegelhalter et al., 2004a).

The purpose of the current research was to re-evaluate the results of a clinical trial for a control plan for bovine mastitis (Green et al., 2007). A community of priors was incorporated, within a Bayesian context, to represent a spectrum of prior opinions of clinicians. The aim of the research was to assess the variability in clinical interpretation that could arise from veterinary surgeons with different prior viewpoints.

## 2 Method

### 2.1 Description of the original clinical trial

The original research comprised a randomised clinical trial for a mastitis control plan on 52 dairy herds and has been described in detail in Green et al. (2007). A brief outline of the methods and results is presented here. In January 2004, a database administered by National Milk Records (NMR, Chippenham, UK) was used to identify and randomly select dairy herds with a recorded incidence rate of clinical mastitis >35 cases per 100 cows during the previous 12 months. Selection was made from herds situated throughout England and Wales. Herds were randomly allocated to one of two groups. The first group had an intervention—a mastitis control plan implemented (this was a holistic control scheme devised from research literature) whilst the second group were treated as control herds. The first stage of the control plan was to assess the patterns and types of mastitis in each herd. The second stage was to compare existing farm control measures with those in the control plan to highlight the measures not used by the farmer. A level of importance was attached to each control measure to determine a priority for implementation and a set of up to 20 final recommendations were made to each farmer in the intervention group. Compliance with the control plan was measured and estimated as the proportion of recommendations made that were actually implemented by the farmer during the 1 year study period. A simple categorisation was used for compliance, this was a score of one given when less than one-third of recommendations were applied, two when between one and two-thirds were applied and three when greater than two-thirds were applied.

A response variable used to assess efficacy of the control plan was the change in incidence rate of clinical mastitis between year 1 (the 12 months before the intervention was carried out) and year 2 (the 12 months following the date of intervention) expressed as a proportion of the year 1 incidence rate of clinical mastitis. The null hypothesis for the study was that there would be no difference in the change in incidence rate of clinical mastitis between intervention farms that implemented the plan (*n* = 26) and control farms that did not (*n* = 26). The alternative hypothesis was that there would be a difference between treatment groups in the mean proportional change of clinical mastitis of ≥0.20 and conventional sample size estimates were undertaken with a power set at 0.8 and significance probability at 0.05. The original analysis was conducted within a frequentist framework and the relevant results were as follows:

1. Proportional change in incidence rate of clinical mastitis for intervention herds versus control herds = −0.20 (SE = 0.09), *p* < 0.05.
2. Proportional change in incidence rate of clinical mastitis for compliance score 3 herds versus control herds = −0.39 (SE = 0.14), *p* < 0.01.

### 2.2 Bayesian models

The two models from which these results were calculated in the original study were replicated using the same data, and placed within a Bayesian framework with specification of prior distributions for model parameters. Model parameter posterior distributions were estimated using Markov chain Monte Carlo using the WinBUGS software package (Version 1.4, Spiegelhalter et al., 2004b).

The models considered for the current analysis were

Model 1:
  $$\begin{array}{l} y_{i}=\beta_{0}+\beta_{1}\text{I}{\text{F}}_{i}+\beta_{2}\text{IRCMyr}1_{i}+e_{1i}\\ e_{1i}∼N(0,\sigma_{e1}^{2}) \end{array}$$
Model 2:
  $$\begin{array}{l} y_{i}=\beta_{0}+\beta_{3}C3_{i}+\beta_{4}C2_{i}+\beta 5C1+\beta_{6}\text{IRCMyr}1_{i}+e_{2i}\\ e_{2i}∼N(0,\sigma_{e2}^{2}) \end{array}$$

where *y*_*i*_: proportional change in incidence rate of clinical mastitis in herd *i*; *β*_0_: model intercept; IF_*i*_: covariate to identify intervention farms,; *β*_1_: coefficient representing the mean proportional change in incidence rate of clinical mastitis for intervention herds compared to control herds.; IRCMyr1_*i*_: covariate to account for starting incidence rate of clinical mastitis in herd *i*; *β*_2_ and *β*_6_: coefficients for year 1 IRCM; C3_*i*,_ C2_*i*_, C1_*i*_: covariates for herds of compliance categories 3, 2 and 1 respectively compared to control herds.; *β*_3_, *β*_4_ and *β*_5_: coefficients for compliance categories 3, 2 and 1 respectively.; *e*_1*i*_ and *e*_2*i*_: residual terms to reflect unexplained variation between herds with variance $\sigma_{e1}^{2}$ and $\sigma_{e2}^{2}$ in Models 1 and 2, respectively.

Models were run with three Markov chains and the effect of different chain starting values on model parameters was investigated but not found to influence posterior estimates. Model convergence was examined using informal visual assessment of the chains (Gilks et al., 1996) and the Gelman–Rubin convergence diagnostic (Brooks and Gelman, 1998). All of the MCMC analyses reported in the current paper used a burn-in of at least 2000 iterations and all models converged well ahead of the end of this burn-in. Analysis was then based on an additional 20,000 iterations.

### 2.3 Prior distributions

A range of Gaussian prior distributions were assessed for the fixed effect parameters *β*_0_, *β*_2_, *β*_4_, *β*_5_ and *β*_6_, to investigate their influence on *β*_1_ (Model 1) and *β*_3_ (Model 2). Gaussian distributions were investigated with a mean of 0.5, 0, or −0.5 and a variance of 10,000 or 0.25. No substantive differences were identified in model results and the distribution (mean = 0, variance = 10,000) was used for the final models. Several alternative priors for the distributions of *e*_1*i*_ and *e*_2*i*_ were investigated (Uniform (0, 5) or Uniform (0, 1) for the standard deviations or inverse Gamma (0.01, 0.01) for the variances) but the choice had little effect on the other parameter estimates and Uniform (0, 5) priors were used in the final models. Six different prior distributions were incorporated for the coefficients of interest, *β*_1_ (Model 1) and *β*_3_ (Model 2); the aim was to choose priors that would cover a realistic and reasonable range of clinical opinion and that could represent views sensibly held by clinicians. This community of priors is described in Tables 1 and 2.

### 2.4 Financial evaluation

To further elucidate possible differences in interpretation of the clinical trial data between clinicians with different prior beliefs, a financial evaluation was carried out as follows. Models were extended to include a financial gain (or loss) attributed to the anticipated change in clinical mastitis (£s per cow in the herd per year) conditional on the clinical trial data and the prior distributions. The estimated cost of a case of clinical mastitis was based on a recent publication of disease costs in UK dairy herds (Esslemont and Kossaibati, 2002). The mean estimated cost per case was a combination of treatment costs (including veterinary time), herdsman time, discarded milk, reduced subsequent milk yield, severity of disease and risk of culling or death (Esslemont and Kossaibati, 2002). Milk price has recently increased in the UK, however, and there is currently some variation between farms. Therefore a distribution for milk price was included in the calculation, based on current prices, with a mean of £0.25/l and standard deviation of £0.01/l. Other financial values remained as originally reported (Esslemont and Kossaibati, 2002). The resultant cost of a case of clinical mastitis was normally distributed with mean £212.30 and standard deviation £5.44. The financial gain anticipated from implementing the control plan on a herd with an assumed incidence rate of clinical mastitis of 0.5 cases per cow per year (the approximate mean value for UK farms (Bradley et al., 2007)) was estimated from the posterior (predictive) distribution of *β*_1_ (the proportional reduction in clinical mastitis estimated from Model 1) and the cost per case of clinical mastitis:

$$\text{Anticipated}\;\text{financial}\;\text{gain}\;(£s\;\text{per}\;\text{cow}\;\text{in}\;\text{the}\;\text{herd}\;\text{per}\;\text{year})=\beta_{1}\times \text{cost}\;\text{of}\;\text{mastitis}\;(N(\text{mean}=212.30,\text{variance}=25.59))\times 0.5$$

The distribution of the anticipated financial gain was estimated for each prior distribution of *β*_1_ (Table 1), using MCMC, by evaluating 20,000 iterations after model convergence. To obtain an estimate for the financial returns anticipated in a fully complying herd, the procedure was repeated using *β*_3_ and Model 2.

The probabilities of obtaining financial returns greater or equal to specified financial levels (between £0 and 50 per cow in the herd per year) were estimated for each prior distribution of *β*_1_ and *β*_3_. This was carried out within the MCMC procedure as follows: at each iteration, an indicator variable was set to 1 when the model predicted the financial return was greater than a specified value and otherwise to 0. The mean value of this indicator over the 20,000 iterations after convergence provided an estimate of the probability of exceeding the specified financial return, a method similar to that described for Monte Carlo P values (Marshall and Spiegelhalter, 2003).

### 2.5 Investigations of posterior distributions and simulations of increased trial sizes

The heterogeneity in the posterior distributions of *β*_1_ (Model 1) was further explored by including a wider variety of combinations of values for both the prior mean (range 0 to −0.3) and prior standard deviation (range 0.001–100). Modelling procedures were as described above, and the mean posterior values for *β*_1_, for different values of prior mean and standard deviation, were displayed graphically.

MCMC simulations were carried out to predict the possible effect of increasing the size of the original clinical trial on the inferences of clinicians with originally sceptical or originally enthusiastic prior beliefs. Simulations of larger sized trials were conducted by replicating the original dataset by a factor of two, three and four times. Therefore, the distribution of the data and model coefficients remained the same but the uncertainty associated with parameter estimates was reduced. Posterior distributions for *β*_1_ were estimated for each simulated trial of increased size, incorporating either a sceptical or enthusiastic prior distribution for *β*_1._ The probability of anticipated financial changes attributed to changes in clinical mastitis were calculated for each simulated trial, as described in Section 2.4, and displayed graphically.

## 3 Results

### 3.1 Posterior distributions of *β*_1_ and *β*_3_

The posterior distributions of *β*_1_ from Model 1 are presented in Table 3. With the inclusion of the vague prior distribution for *β*_1_, the resulting posterior distribution had very similar characteristics to the original frequentist estimates for *β*_1_. The probabilities for an anticipated financial saving from a reduced incidence of clinical mastitis were 0.85 for a return of at least £10 per cow in the herd and 0.54 for a return of at least £20 per cow in the herd. The sceptical prior resulted in a posterior mean estimate for *β*_1_ of −0.10, half way between the sceptical prior mean and the original frequentist estimate for *β*_1,_ indicating that a clinician with this sceptical viewpoint would effectively discount the clinical trial results by approximately 50% compared to the frequentist interpretation of the data. The very sceptical prior was altered very little by the trial data, the posterior mean of −0.04 being one-fifth of the original frequentist estimate for *β*_1_ and thus these data would make only a small impact on a clinician with such a large degree of scepticism. The cautious sceptic (Table 1), would be more convinced by the trial results, the posterior mean estimate for *β*_1_ being −0.13. This was similar to the posterior mean of −0.15 estimated with a prior belief that *β*_1_ was at the mid-point between the sceptical and enthusiastic priors. The enthusiastic prior resulted in a similar mean posterior estimate to the vague prior (−0.20), but with a reduced standard deviation (0.07) indicating a greater certainty in the posterior mean if this prior view was held. The posterior mean for *β*_1_ estimated using a very enthusiastic prior was −0.28 and thus remained closer to this prior distribution mean than the original frequentist mean estimate.

The characteristics for the posterior distributions of *β*_3_ estimated in Model 2 are presented in Table 4. Similar patterns were identified between the different choices of prior as for *β*_1_. It was particularly notable that whilst the sceptical prior resulted in a posterior distribution indicating a reasonably large anticipated reduction in clinical mastitis (posterior mean−0.21, standard deviation = 0.11), the original trial data would be insufficient to convince clinicians with views represented by the very sceptical prior distributions, that the control plan would be very effective, even in herds that fully complied (posterior mean = −0.04, standard deviation = 0.05).

### 3.2 Financial evaluations

**Fig. 1:**
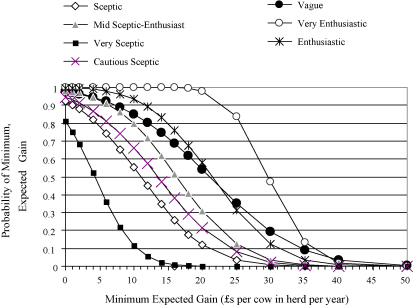
An illustration of the probability of the anticipated overall financial gains from implementing the mastitis control plan estimated from Model 1, conditional on specified prior distributions (Table 1) and the clinical trial data.

**Fig. 2:**
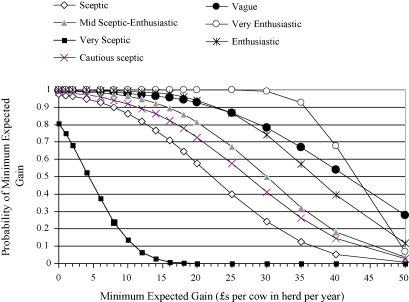
An illustration of the probability of anticipated financial gains from implementing the mastitis control plan on farms that fully comply, estimated from Model 2, conditional on the specified prior distributions (Table 2) and the clinical trial data.

The anticipated financial returns estimated from Models 1 and 2 are presented in Figs. 1 and 2. Clinicians with beliefs represented by any of the prior distributions, except the very sceptical category, would anticipate a gain of ≥£10 in this “average” herd that undertook the control plan with a probability of ≥0.50. The posterior probability of a gain ≥£10 varied from 0.55 for the sceptical prior distribution to 0.99 for the very enthusiastic prior.

There was considerable variation in the posterior probability of achieving a gain of at least £20 per cow in the herd dependent on the different prior distributions. The very sceptical prior resulted in a posterior probability of virtually zero whereas the vague or enthusiastic priors resulted in a posterior probability greater than 0.50. A clinician with a view represented by the very enthusiastic prior view would anticipate a gain ≥£20 as being almost certain with a probability approaching 1.0.

Taken as a whole, results from Model 1 (Fig. 1) demonstrated that the probability of different financial gains varied greatly with different prior distributions. This variability indicates that, in light of the clinical trial data, clinicians could differ widely in their approach to implementing the plan, some anticipating considerably more financial return than others.

The pattern of results was broadly similar when herd compliance with the control plan was considered in Model 2 (Fig. 2), although with higher anticipated financial returns. Parameter estimates revealed that clinicians with beliefs represented by any of the prior distributions, except the very sceptical prior, would anticipate a 0.50 probability of a gain of more than £22 per cow in a herd that undertook and fully complied with the control plan, compared to a non-participant. However, there was considerable variability in the anticipated probability of achieving minimum gains in the region of £20–40 per cow in the herd (Fig. 2), dependent on prior clinical beliefs, again indicating that the trial data are likely to lead to different clinical interpretations dependent upon different initial viewpoints.

### 3.3 Further assessment of the prior distribution characteristics

**Fig. 3:**
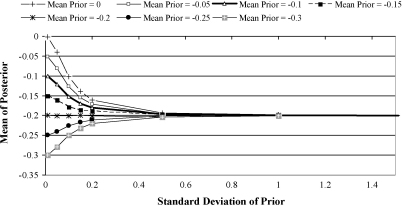
Graphical illustration of the variation of the prior mean and standard deviation, and the posterior mean of *β*_1,_ estimated from Model 1, conditional on the clinical trial data.

As illustrated in Fig. 3, the mean posterior value of *β*_1_ varied to a large extent with both the mean and standard deviation of the prior distribution of *β*_1_. The posterior mean tended towards the value of the prior mean as the prior standard deviation decreased and tended towards −0.20 as the prior standard deviation increased. A given posterior mean value (for example −0.15) could result from a prior distribution with a mean close to that posterior mean (e.g. −0.15) with a relatively small standard deviation, or from a prior mean closer to zero, and a relatively larger standard deviation.

### 3.4 Simulations of increased sized trials

**Fig. 4:**
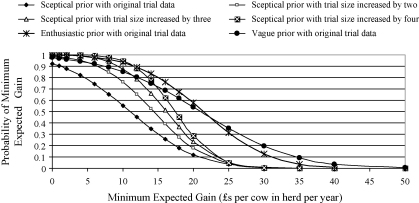
An illustration of the probability of the anticipated overall financial gains from implementing the mastitis control plan estimated from Model 1, using the original trial data and three simulated trials of increased size that incorporated the sceptical prior (Table 1).

**Fig. 5:**
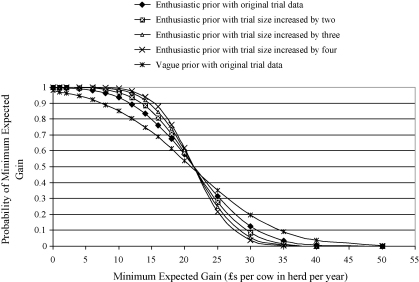
An illustration of the probability of the anticipated overall financial gains from implementing the mastitis control plan estimated from Model 1, using the original trial data and three simulated trials of increased size that incorporated the enthusiastic prior (Table 1).

Simulations of anticipated financial returns with different theoretical sizes of trial are summarised in Fig. 4. Simulations using the sceptical prior distribution demonstrated that the anticipated financial gain gradually increased (curves moved to the right) and the variation of the posterior distribution decreased (curves became steeper) as trial size increased. However, the increase in anticipated financial gain became proportionately less as the new data became less influential in comparison to the existing information. With a clinical trial four times the size of the original, an initial sceptic would consider that there was a comparable probability (0.93) of a financial gain of at least £10 per cow in the herd, to an initial enthusiast after the original sized clinical trial. However, after a study size four times larger than the original, an initial sceptic would still attribute a lower probability (0.28) than the initial enthusiast would attribute after the original study (0.58), that a minimum gain of £20 per cow would be achieved. Simulations of increased trial sizes using the enthusiastic prior as the starting point (Fig. 5) demonstrated that anticipated financial gains would change very little with the increased information, although the clinician with this original belief would become more certain of their anticipated financial return with the additional information.

## 4 Discussion

The results suggest that a clinician's prior view will make a fundamental impact on how the clinical trial data for this mastitis control plan are interpreted. An initial sceptic, when presented with this evidence would anticipate a reduction in clinical mastitis approximately half that of the initially enthusiastic clinician. This translates into large differences in anticipated financial gains from reduced clinical mastitis, often in the region £5 and £20 per cow in the herd (Figs. 1 and 2). The differences in clinical interpretation and anticipated financial return dependant on prior viewpoints are of a magnitude that would make important material differences in practice and are likely to influence the assessment of when the plan is cost effective and therefore recommended. The results also illustrate why a conventional ‘significant’ result may provide an insufficient strength of evidence to change the clinical approaches of some more sceptical clinicians.

The costs of implementing this mastitis control plan will vary between herds depending on the management changes required (Green et al., 2007), but the annual costs are likely to be in the region £5 to £50 per cow in the herd. For a 100 cow herd this equates to a variation in total implementation costs of £500 to £5000 per annum, reflecting, for example, differences between small changes to current management or a capital investment with repayment over several years. Therefore differences in anticipated financial return of £5 to £20 per cow, depending on a clinicians view, will be important in determining whether the plan is implemented, and therefore could lead to different clinical decisions in the same farm circumstances.

An individual clinician's decision to change their approach to disease control, however, is complex and will depend on a variety of psychological factors, as well as the perceived cost or health benefit. A rational decision has been defined as one that meets four criteria (Hastie and Dawes, 2001): it is based on the decision makers current state (physiological, psychological, financial, social and emotional), on the possible consequences of the choice, on the logical probability of different outcomes and is adapted according to the value placed on each possible outcome. Therefore, the situation, cognitive dispositions and personality of a clinician will influence how decisions are made (Crosskerry, 2005) and this means that individual veterinary clinicians with the same prior beliefs and presented with the same research evidence may make different judgements. Factors such as the veterinary surgeon-client relationship, the farm situation (such as the financial state), the value attributed to non-financial benefits (such as cow welfare) will influence decision-making alongside the current mastitis situation and anticipated return on investment from implementing the plan. Whilst different prior beliefs can greatly affect the interpretation of a single piece of research evidence, more research would be useful to investigate and quantify other aspects of the decision processes in farm animal health.

Results from the current study confirm the importance of both the magnitude (location) of a prior belief and the certainty with which the view is held (Fig. 3). When the certainty of a prior belief declines, the influence of the data increases and the posterior mean tends towards the likelihood of the data. Therefore, to assess a clinician's final belief, in light of new evidence, it is important to establish not only the central location of their prior view but also the certainty with which it is held, and methods have been described to elicit these quantities (O’Hagan et al., 2006). The graph of different prior distribution means and standard deviations in relation to subsequent posterior distribution means (Fig. 3) illustrates prior distribution standard deviations varying from very small (0.01) to relatively large (2.0). In reality, such extreme clinical views would be unlikely. It seems improbable that a clinician would have a belief with a 95% credibility interval as small as ±0.02 around the estimated mean (a change in mastitis of only ±2% of the initial level). Similarly, a prior standard deviation of 1.00 would mean that a clinician was so uncertain that there was a 95% credibility interval of approximately ±2.00 around the mean effect (a change in mastitis of ±200% of the initial level), this would appear to be greater than could be reasonably anticipated. It is likely that most clinical prior beliefs would have a standard deviation in the region 0.05–.3 and thus the region of the graph (Fig. 3) within this range probably contains the most realistic representation of the relationship between plausible clinical prior distributions and the posterior mean.

The vague prior distributions used in these analyses for the fixed effects *β*_1_ and *β*_3_ are often described as “non-informative” or “reference” priors (Spiegelhalter et al., 2004a) and often will result in a posterior distribution with characteristics similar to the frequentist estimates (Gurrin et al., 2000). However, it is hard to ascribe a realistic, rational clinical meaning to this prior distribution since such an uninformed view would seem to be almost impossible to be held by a clinician. Therefore, whilst it is useful in terms of allowing the data to have “an overriding influence” on the posterior distribution, it is of limited value when trying to evaluate the likely interpretation of clinicians in the field. Since Bayesian philosophy essentially concerns the updating of personal beliefs in the light of new evidence, the use of vague, unrealistic prior distributions has understandably been questioned (Senn, 2007).

The value of assessing the effect of sceptical priors in clinical trials is illustrated in the current study by the important clinical differences that would arise in interpretation of these mastitis trial data. Sceptical priors are also useful to estimate sample sizes for clinical trials (Chaloner and Rhame, 2001) and if scepticism is known to be common, then it is important to design a study that can convince sufficient members of the relevant population. In the current study, simulations of clinical trials of increased size identified that with more data, a clinician with an initial sceptical view would gradually become more convinced of the efficacy of the control plan. If such a sceptical view is very prevalent in the population of veterinary surgeons, then larger studies would be important to provide sufficient evidence to convince these clinicians that the control plan could be worthwhile.

Bayesian methods are particularly useful to calculate probabilities that may be extrapolated directly to clinical practice (Burton et al., 1998; Lilford et al., 1995) and this research provides an illustration in veterinary medicine. Although the testing of a variety of prior opinions in Bayesian analyses has been strongly recommended to demonstrate how new data would add to the range of currently held views (Koch, 1991; Hughes, 1991; Spiegelhalter et al., 2004a) this type of analysis has been rarely reported in the medical or veterinary literature. However, it is necessary to understand clinical inferences and the decision-making process, beyond simply providing new evidence, if research is going to have a widespread impact on animal health. This is particularly true in veterinary medicine in which there is little overall national strategy for many diseases of dairy cows and in which many decisions on herd health are taken by individual practitioners rather than being set by a general health policy. Research to quantify the population structure of veterinary beliefs for mastitis control and other aspects of herd health management would be very welcome to improve the understanding of the diversity of clinical approaches exhibited by veterinary surgeons. Knowledge of the degree and distribution of scepticism and enthusiasm would help to inform future research both in terms of research areas most needed and the strength of evidence that would be necessary to convince clinicians that changing approaches could be beneficial.

## Figures and Tables

**Table 1:** Descriptions of the prior distributions for *β*_1_ incorporated into Model 1 to represent a range of clinical viewpoints on the probable effectiveness of the mastitis control plan.

Name of prior	Distribution (mean, SD)	Description of view represented
Vague	Normal (0, 100)	No view or ability to make a choice as to what the likely parameter values could be and therefore prepared to encompass a very large possible range.
Very sceptic	Normal (0, 0.05)	A mean effect size of 0 with a 2.5% probability that the effect size could be a reduction in mastitis more than −0.10
Sceptic	Normal (0, 0.10)	A mean effect size of 0 with a 2.5% probability that the effect size could be a reduction in mastitis more than −0.20
Mid sceptical enthusiastic	Normal (−0.1, 0.10)	A mean effect size of −0.10 with a 15% probability that the effect size could be a reduction in mastitis greater than −0.20 or less than 0.
Cautious sceptic	Mixture: Normal (0, 0.10) × 0.80 + Normal (0, 0.5) × 0.20	A weighted mixture of a sceptical prior (80% probability) and a more cautious prior (20% probability) to reflect a sceptical who is prepared to allow a 20% probability that their sceptical belief is incorrect and to widen the standard deviation of the effect size by a factor of five.
Enthusiastic	Normal (−0.2, 0.10)	A mean effect size of −0.20 with a 2.5% probability that the effect size could be a reduction in mastitis less than 0.
Very enthusiastic	Normal (−0.3, 0.05)	A mean effect size of −0.30 with a 2.5% probability that the effect size could be a reduction in mastitis less than −0.20.

**Table 2:** Descriptions of the prior distributions for *β*_3_ incorporated into Model 2 to represent a range of clinical viewpoints on the probable effectiveness of the mastitis control plan.

Name of prior	Distribution (mean, SD)	Description of view represented
Vague	Normal (0, 100)	No view or ability to make a choice as to what the likely parameter values could be and therefore prepared to encompass a very large possible range.
Very sceptic	Normal (0, 0.05)	A mean effect size of 0 with a 2.5% probability that the effect size could be a reduction in mastitis more than −0.10
Sceptic	Normal (0, 0.15)	A mean effect size of 0 with a 2.5% probability that the effect size could be a reduction in mastitis more than −0.30
Mid sceptical enthusiastic	Normal (−0.15, 0.15)	A mean effect size of −0.10 with a 15% probability that the effect size could be a reduction in mastitis greater than −0.20 or less than 0.
Cautious sceptic	Mixture: Normal (0, 0.15) × 0.80 + Normal (0, 0.75) × 0.20	A weighted mixture of a sceptical prior (80% probability) and a more cautious prior (20% probability) to reflect a sceptical who is prepared to allow a 20% probability that their sceptical belief is incorrect and to widen the standard deviation of the effect size by a factor of five.
Enthusiastic	Normal (−0.3, 0.15)	A mean effect size of −0.30 with a 2.5% probability that the effect size could be a reduction in mastitis less than 0.
Very enthusiastic	Normal (−0.4, 0.05)	A mean effect size of −0.40 with a 2.5% probability that the effect size could be a reduction in mastitis less than −0.30.

**Table 3:** Posterior distributions of *β*_1_ estimated from Model 1 conditional on the specified prior distributions and the clinical trial data.

Name of prior	Prior distribution	Posterior distribution	
	Mean (SD)	Mean (SD)	95% credibility interval
Vague	0 (100)	−0.20 (0.10)	−0.39 to 0.00
Very sceptic	0 (0.05)	−0.04 (0.05)	−0.13 to 0.05
Sceptic	0 (0.10)	−0.10 (0.07)	−0.25 to 0.04
Mid sceptical enthusiastic	−0.10 (0.10)	−0.15 (0.07)	−0.30 to −0.01
Cautious sceptic	0 (0.13)	−0.13 (0.08)	−0.28 to 0.03
Enthusiastic	−0.20 (0.10)	−0.20 (0.07)	−0.34 to −0.06
Very enthusiastic	−0.30 (0.05)	−0.28 (0.04)	−0.37 to −0.19

**Table 4:** Posterior distributions of *β*_3_ estimated from Model 2 conditional on the specified prior distributions and the clinical trial data.

Name of prior	Prior distribution	Posterior distribution	
	Mean (SD)	Mean (SD)	95% Credibility interval
Vague	0 (100)	−0.39 (0.14)	−0.67 to −0.11
Very sceptic	0 (0.05)	−0.04 (0.05)	−0.13 to 0.05
Sceptic	0 (0.15)	−0.21 (0.11)	−0.41 to 0.00
Mid sceptical enthusiastic	−0.15 (0.15)	−0.28 (0.10)	−0.49 to −0.07
Cautious sceptic	0 (0.19)	−0.26 (0.12)	−0.48 to −0.03
Enthusiastic	−0.30 (0.15)	−0.35 (0.10)	−0.55 to −0.15
Very enthusiastic	−0.40 (0.05)	−0.40 (0.05)	−0.49 to −0.31
